# Acquisition of toxin-encoding lysogenic bacteriophage elements enhances the virulence of pandemic *Streptococcus pyogenes* M1_UK_

**DOI:** 10.1128/iai.00503-25

**Published:** 2026-02-09

**Authors:** Juan Manuel Díaz, Jasmine E. J. Wells, Amanda C. Marple, Blake A. Shannon, Aanchal Rishi, Irene Martin, Allison McGeer, Matthew A. Croxen, Gregory J. Tyrrell, Mark J. Walker, Stephan Brouwer, John K. McCormick

**Affiliations:** 1 Department of Microbiology and Immunology, University of Western Ontario 6221, London, Ontario, Canada; 2 Australian Infectious Diseases Research Centre and Institute for Molecular Bioscience, The University of Queensland 589067 , St. Lucia, Queensland, Australia; 3 National Microbiology Laboratory, Public Health Agency of Canada, Winnipeg, Manitoba, Canada; 4 Department of Microbiology, Sinai Health System 7982, Toronto, Ontario, Canada; 5 Department of Laboratory Medicine and Pathobiology, University of Toronto 233837, Toronto, Ontario, Canada; 6 Department of Laboratory Medicine and Pathology, Faculty of Medicine and Dentistry, University of Alberta 536883, Edmonton, Alberta, Canada; 7 Alberta Precision Laboratories-Public Health Laboratory 113196 , Edmonton, Alberta, Canada; 8 Li Ka Shing Institute of Virology, University of Alberta 600166, Edmonton, Alberta, Canada; 9 Women and Children's Health Research Institute, University of Alberta 3158, Edmonton, Alberta, Canada; St Jude Children's Research Hospital, Memphis, Tennessee, USA

**Keywords:** bacteriophages, group A *Streptococcus*, invasive disease, M1_UK_, scarlet fever, *Streptococcus pyogenes*

## Abstract

Multiple countries have observed an alarming increase in scarlet fever cases, and invasive infections often associated with a new sublineage of *Streptococcus pyogenes* known as M1_UK_. M1_UK_ strains express increased levels of the streptococcal pyrogenic exotoxin A (SpeA) superantigen, and here we compare the virulence characteristics of this sublineage with the circulating M1_global_ strain. We obtained contemporary Canadian M1_UK_ isolates, and genome sequencing revealed that some M1_UK_ strains had acquired additional DNAse- and superantigen-encoding prophage elements, as well as an isolate with a mutation in *covS*. Five *S*. *pyogenes* strains were chosen for functional experiments, including 5448 (M1_global_ strain), M1_UK_350 (a “typical” M1_UK_ strain), M1_UK_162 (M1_UK_ strain containing a mutation in the *covS* gene), M1_UK_362_ΦSP1380.vir_ (M1_UK_ strain containing a prophage element encoding the *spd1*, *speC*, and *ssa* genes), and M1_UK_155_Φ370.1_ (M1_UK_ strain containing a prophage element encoding the *spd1* and *speC* genes). Exoprotein profiles demonstrated that all M1_UK_ background strains had enhanced production of the SpeA superantigen relative to *S. pyogenes* 5448. Furthermore, strains that had acquired the additional prophage elements showed enhanced activation for human T cells, although cytotoxic activity, adhesion capacity, and DNA degradation were not detectably different. Using a “humanized” superantigen-sensitive HLA-transgenic mouse infection model, the M1_UK_162 *covS* mutant, and both M1_UK_362_ΦSP1380.vir_ and M1_UK_155_Φ370.1_ strains each demonstrated increased severity during experimental skin infection compared to 5448 and M1_UK_350. These findings indicate that circulating M1_UK_ background strains continue to acquire additional prophage-encoded virulence factors, or hypervirulent *covS* mutations, and that these genetic alterations may contribute to increase severity of human infections.

## INTRODUCTION

*Streptococcus pyogenes* (commonly referred to as the group A *Streptococcus*) is a human-restricted bacterial pathogen responsible for common infections such as pharyngitis and impetigo, invasive infections including necrotizing fasciitis and myositis, and the toxin-mediated diseases scarlet fever and streptococcal toxic shock syndrome. Additionally, post-infection sequelae resulting from repeated infections can lead to autoimmune rheumatic heart disease (1). Although *S. pyogenes* primarily exists in an asymptomatic carriage state, invasive *S. pyogenes* infections and complications from rheumatic heart disease are responsible for considerable human mortality, with at least ~500,000 deaths each year (2, 3).

*S. pyogenes* is serotyped according to the variability of the 5′ end of the M protein gene (*emm*). During the mid-1980s, an *emm1* genotype (now commonly referred to as “M1_global_”) emerged as one of the most frequent M-types isolated from invasive infections in high-income countries (4), and this emergence has been attributed to the acquisition of a prophage encoding the streptococcal pyrogenic exotoxin A (SpeA) superantigen and the DNase Sda1, and a genetic recombination event that resulted in increased expression of the streptolysin O operon (5–7). More recently, a global increase in infections has been caused by the emergence of an *emm1* sublineage known as M1_UK_ that is characterized by 27 core genome single-nucleotide polymorphisms (SNPs) compared with M1_global_ strains, and a ~10-fold increased expression of SpeA (8). The increase in SpeA expression was traced to a single SNP located upstream of the *ssrA* gene, resulting in increased downstream transcription of *speA* (9). SpeA is understood to be a key *S. pyogenes* virulence factor involved in the streptococcal toxic shock syndrome (10), although this superantigen can also promote experimental upper respiratory tract infections (11), and can furthermore induce suppression of immunoglobulin production during recurrent tonsillitis in humans (12, 13). Thus, enhanced SpeA expression contributes to the severity of invasive infections but may also be linked to both a fitness advantage for carriage and transmission.

The remarkable expansion of *S. pyogenes* M1_UK_ and its replacement of classical circulating strains warrants a multidisciplinary approach to understanding and controlling this important public health concern. Here, we phenotypically characterized four Canadian M1_UK_ strains in comparison to the well-studied *S. pyogenes* 5448 M1_global_ strain using both *in vitro* assays and an *in vivo* humanized superantigen-sensitive mouse skin infection model. These four M1_UK_ strains included a “typical” M1_UK_ strain, a strain with an inactivating mutation in the histidine kinase sensor *covS* (control of virulence) gene, and two additional isolates that had acquired additional virulence factors through the acquisition of lysogenic bacteriophage elements. Our findings suggest that contemporary *S. pyogenes* M1_UK_ isolates continue to evolve to acquire additional bacteriophage-encoded virulence characteristics that likely contribute to enhanced severity of invasive *S. pyogenes* infections.

## RESULTS

### Genotypic and toxin expression differences between M1_UK_ isolates

**Fig 1:**
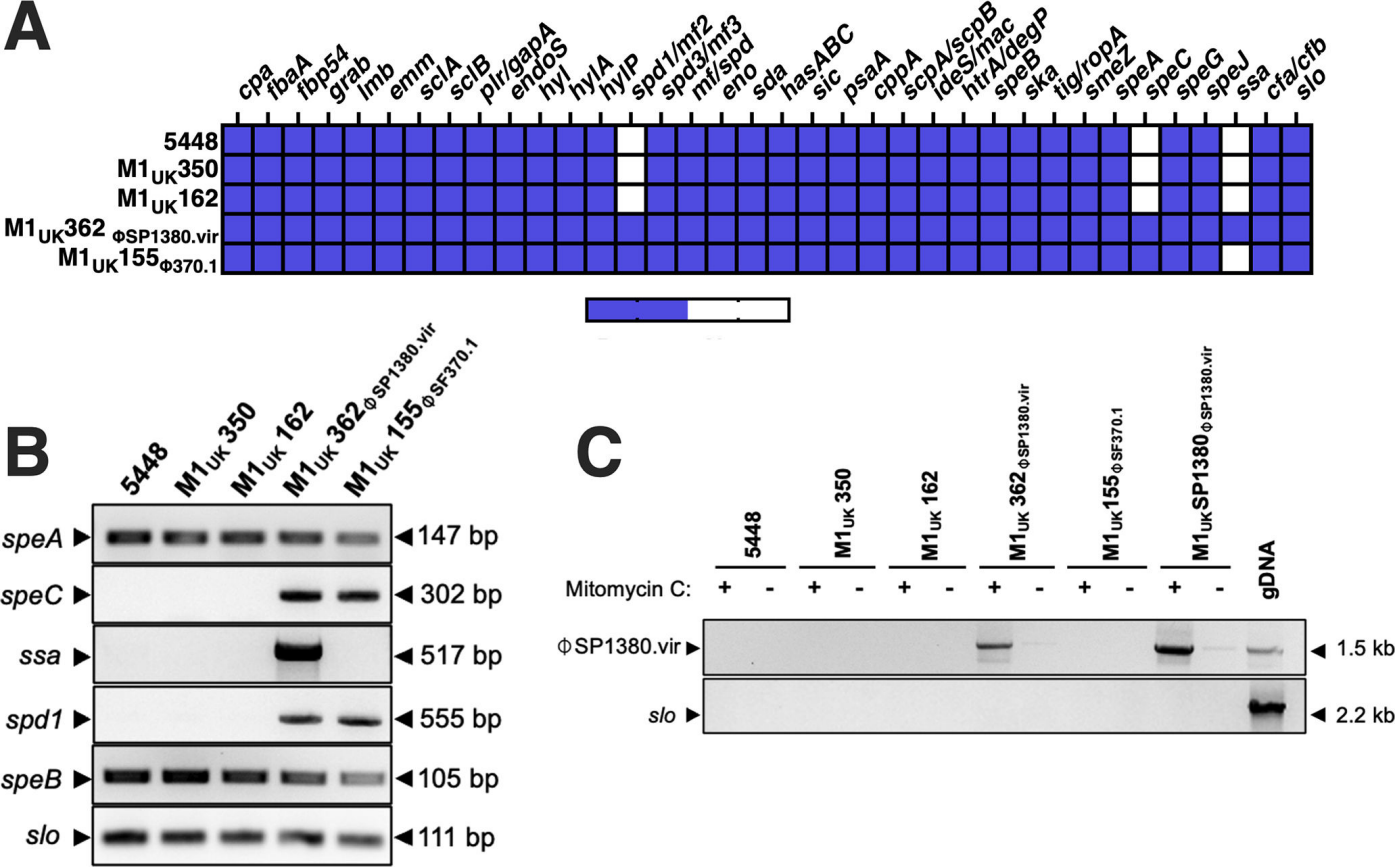
*In silico* and PCR virulence factor gene comparisons of the different *S. pyogenes* strains and detection of ⏀SP1380.vir phages. (**A**) Schematic of virulence factor genes present in the *S. pyogenes* 5448 and M1_UK_ strains used in this study. (**B**) PCR screening of toxin gene carriage, and (**C**) induction of ΦSP1380.vir was assessed by inverted PCR amplification of circularized phage DNA from culture supernatants of *S. pyogenes* following treatment with subinhibitory concentrations of mitomycin C (0.2 µg/mL). Amplification of the *slo* gene served as a negative control to confirm the absence of contaminating chromosomal DNA in phage preparations. The genomic DNA (gDNA) of the Australian M1_UK_ strain SP1380 was used as a positive control.

To characterize and compare the genetic and pathogenic features of the Canadian *S. pyogenes* M1_uk_ clinical isolates, whole-genome sequences were obtained and aligned to the genome of the reference strain MGAS5005 (5). We identified three clinical isolates (M1_UK_155, M1_UK_350, and M1_UK_362) that each contained the 27 characteristic SNPs described in the M1_UK_ strain (8), including the variant within the 5′ region of *ssrA* responsible for enhanced SpeA production (9) (Table 1). From initial experiments evaluating supernatant profiles, we also detected an isolate with an apparent control of virulence (*cov*) mutant phenotype (M1_UK_162), which was subsequently confirmed to contain a Leu_238_→Phe_238_ mutation within the *covS* gene (Table S1). In addition to the characteristic M1_UK_ SNPs, other deletions and SNPs were observed along the complete genomes, highlighting additional genetic variability among the bacterial samples (Table S2). *In silico* analysis of the virulence factors present in each strain revealed a relatively consistent genetic pattern (Fig. 1A); however, the two strains identified as M1_UK_362 and M1_UK_155 exhibited additional virulence factor profiles due to acquisition of the bacteriophages ΦSP1380.vir (encoding *spd1*, *speC*, and *ssa*) or Φ370.1 (encoding *spd1* and *speC*), respectively. For clarity, these latter M1_UK_ strains are referred to as M1_UK_362_ΦSP1380.vir_ and M1_UK_155_Φ370.1_. Consequently, to confirm the differences observed *in silico,* the presence of the virulence factor genes *speA, speC, ssa, spd1, speB,* and *slo* was confirmed by PCR (Fig. 1B). In addition, after performing a bacteriophage induction protocol with mitomycin C, we were able to detect the presence of the ⏀SP1380.vir phage in the supernatant from the M1_UK_362_ΦSP1380.vir_ isolate. (Fig. 1C)

**TABLE 1:** *Streptococcus pyogenes* strains used in this study

Strain	Clinical source	Observations
5448	Necrotizing fasciitis	M1_global_ strain
M1_UK_350	Blood isolate	Classic M1_UK_ strain
M1_UK_162	Blood isolate	M1_UK_ strain containing the *covS* mutation
M1_UK_362	Blood isolate	M1_UK_ strain with acquisition of the phage ΦSP1380.vir
M1_UK_155	Ear fluid	M1_UK_ strain with acquisition of the phage Φ370.1

**Fig 2:**
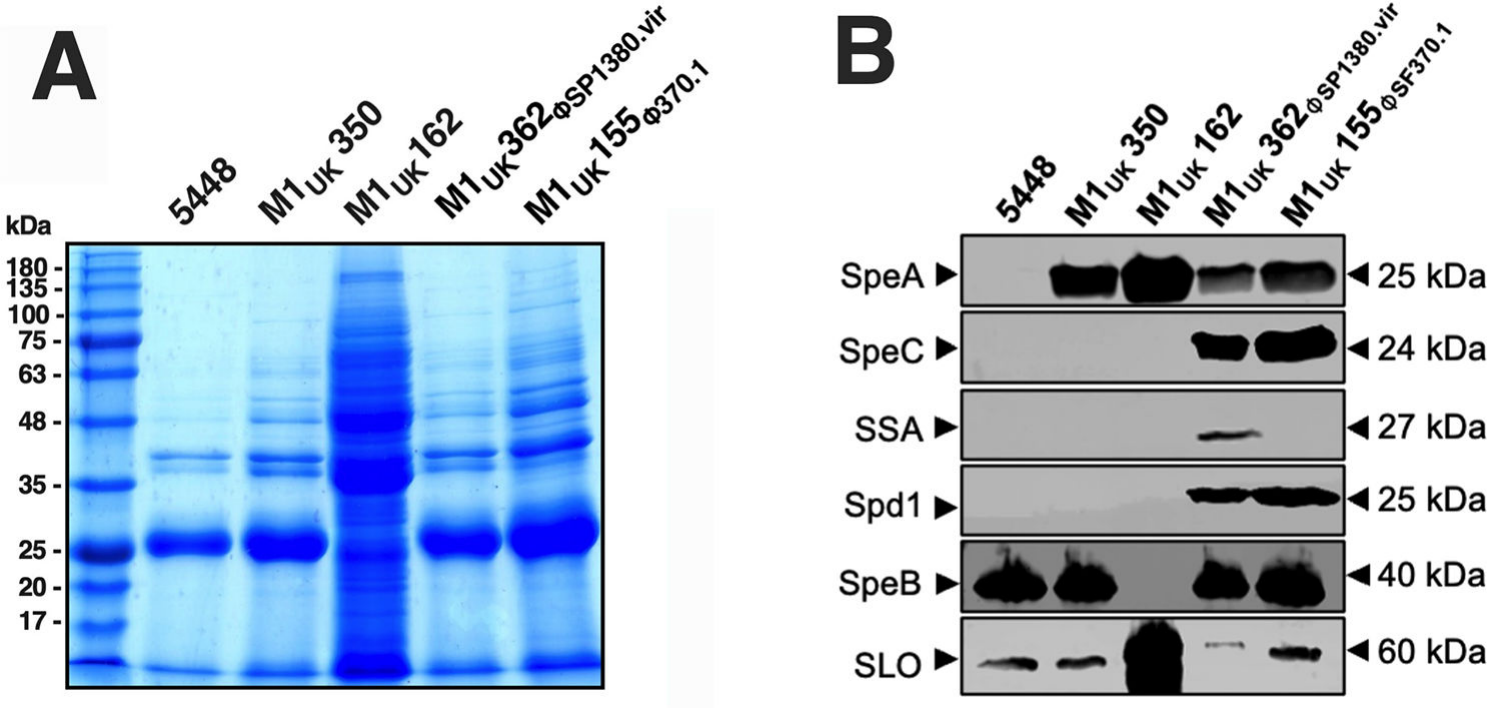
Evaluation of exoprotein profiles of the different *S. pyogenes* M1_UK_ isolates. (**A**) Exoprotein profiles analyzed by SDS-PAGE. (**B**) Western Blot analysis for the three superantigens SpeA, SpeC, and SSA, the DNase Spd1, cysteine protease SpeB, and SLO.

To characterize the secreted protein expression patterns for these strains, bacterial supernatants were collected and evaluated by SDS-PAGE (Fig. 2A) and Western immunoblot (Fig. 2B). From the supernatant protein profiles, the M1_UK_162 strain was dramatically different from the other isolates, which is consistent with the inactivating *covS* mutation and the loss of expression of the SpeB cysteine protease and the overexpression of SLO (14). Importantly, each M1_UK_ isolate displayed the characteristic overexpression of the superantigen SpeA (8), consistent with the SNP located upstream of *ssrA* (9) observed in M1_UK_ strains relative to 5448 (Table S1). Except for the M1_UK_162 *covS* mutant strain, each produced the SpeB protease similar to 5448. As predicted from the acquisition of the additional bacteriophages, the expression of the superantigen SpeC was detected in the M1_UK_362_ΦSP1380.vir_ and M1_UK_155_Φ370.1_ isolates, whereas SSA was only detectably produced by M1_UK_362_ΦSP1380.vir_ (Fig. 2B). These results demonstrate that M1_UK_ isolates continue to exhibit genetic variability due to the identification of additional SNPs, but also can acquire the addition of bacteriophage elements that express additional virulence factors *in vitro*.

### Phenotypical characterization of the *S. pyogenes* M1_UK_ isolates

**Fig 3:**
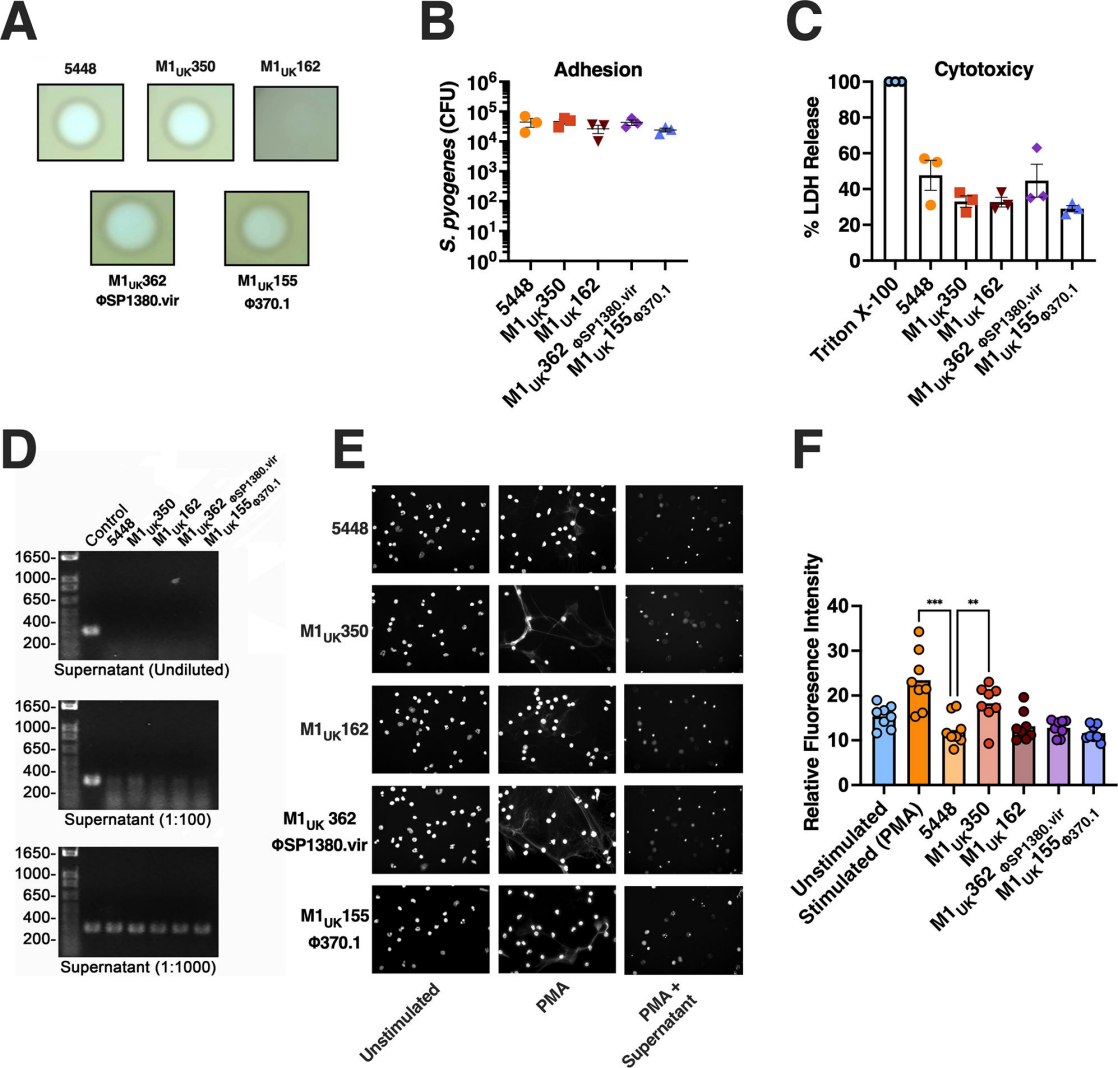
*In vitro* analysis of M1_UK_ phenotypes for proteolytic activity, adhesion, cytotoxicity, and NETs degradation by supernatants. (**A**) The proteolytic activity in the M1_UK_162 CovS mutation strain was absent in comparison with the rest of the strains. The results from the adhesion capacity (**B**) and cytotoxicity (**C**) of the M1_global_ and M1_UK_ strains did not show differences between groups. (**D**) A similar DNA degradation pattern was observed from undiluted and 100-fold diluted supernatants in all strain supernatant samples. (**E**) Human neutrophils from healthy donors were stimulated with 100 nM PMA to generate NETosis, then incubated with 5448 or M1_UK_ strain supernatants, respectively, to evaluate their degradation. (**F**) Analyzing the relative fluorescence of the samples, it was possible to observe a similar behavior in this activity between samples. A total of eight images were analyzed per group, and the differences between groups were assessed using one-way ANOVA with Dunnett’s multiple comparison, using as control the 5448 strain **P < 0.002 and ***P < 0.0002.

Given the *covS* mutation and altered secreted protein profile in the M1_UK_162 strain, the proteolytic activity of all M1_UK_ strains was evaluated using an agar plate-based casein proteolytic assay. As predicted (14), *S. pyogenes* M1_UK_162 had drastically decreased proteolytic activity compared to the other strains, as evidenced by the lack of a zone of inhibition surrounding the isolate, whereas proteolytic activity from the other isolates, including 5448, was similar (Fig. 3A). We also conducted both adhesion assays and cytotoxicity assays using Detroit-562 nasopharyngeal cells, although no differences were detected in either assay as compared with M1_global_ *S. pyogenes* 5448 (Fig. 3B and C). In addition to superantigens, both ΦSP1380.vir and Φ370.1 bacteriophages encode an additional DNase. DNase activity was initially determined by analyzing varying concentrations of bacterial supernatant against PCR products coming from the *speC* amplicon (15). A similar DNA degradation pattern was observed in undiluted and 100-fold diluted supernatants (Fig. 3D). Next, neutrophil extracellular trap (NET) degradation by culture supernatants was evaluated as a further measure of DNase activity (15). Polymorphonuclear cells were isolated from healthy human donors and stimulated with PMA to induce the formation of NETs. Following incubation with the *S. pyogenes* supernatants, we observed a decrease in nuclear staining as well as the complete degradation of NETs by all *S. pyogenes* strains assessed (Fig. 3E). To quantify these observations, relative fluorescence intensity was measured. Samples stimulated with M1_UK_ supernatants showed a similar mean fluorescence intensity to samples stimulated with *S. pyogenes* 5448. In M1_UK_350, a detectable elevation in fluorescence was observed (Fig. 3F).

**Fig 4:**
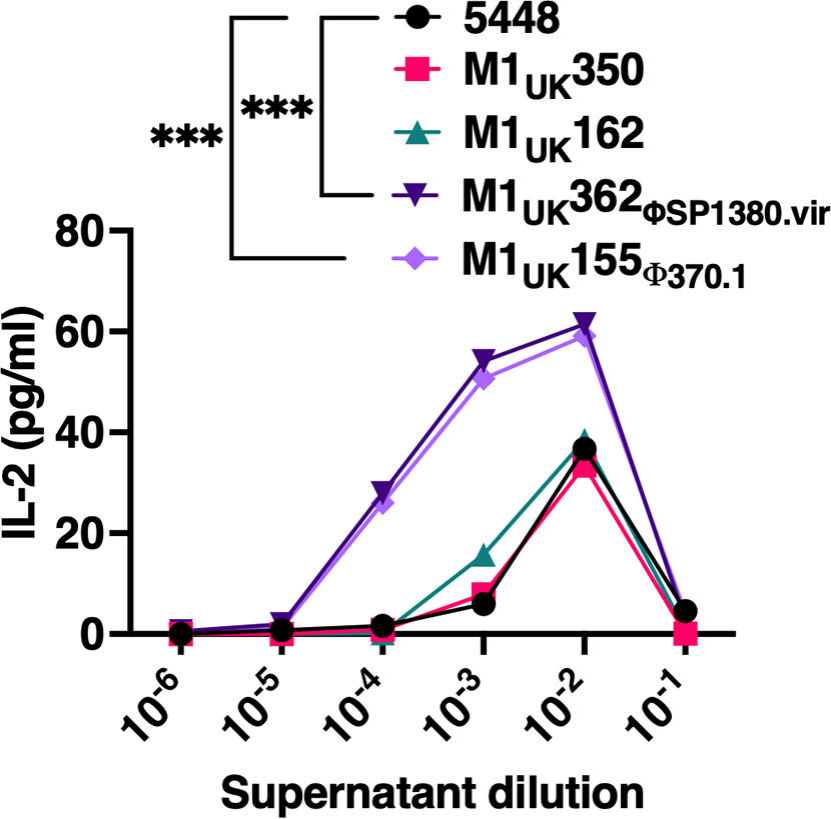
*In vitro* PBMC activation by *S. pyogenes* 5448 and M1_UK_ cell-free bacterial culture supernatants. After isolation, the PBMC stimulation with the supernatant dilutions showed a significant increase in IL-2 production by the M1_UK_362_ΦSP1380.vir_ and M1_UK_155_Φ370_ supernatants. Two-way ANOVA (***P < 0.0002*),* each data point represents the means IL-2 production (*n* = 9). One-way ANOVA, each data point represents a repetition (*n* = 3). Multiple comparison experiments were used as a comparative control for the 5448 strain.

The overexpression of the superantigen SpeA in the M1_UK_ lineage is a phenotypic characteristic among these emergent *S. pyogenes* strains (14). We next quantified the ability of the isolate supernatants to activate T cells using IL-2 production from human peripheral blood mononuclear cells (PBMCs). Although there were no apparent differences between 5448 and the M1_UK_350 and M1_UK_162 isolates, we found a significant increase in IL-2 production following stimulation with the supernatants from *S. pyogenes* M1_UK_362_ΦSP1380.vir_ and M1_UK_155_Φ370.1_ (Fig. 4). The statistical analysis in this assay was performed comparing the highest peaks of IL-2 production (dilution 10^−2^) of each group as more concentrated supernatants are cytotoxic to PBMCs, likely due to the cytolytic toxins (16). These data suggest that apart from the *S. pyogenes* M1_UK_162 *covS* mutant, the different M1_UK_ strains had similar phenotypic characteristics to *S. pyogenes* 5448, although two M1_UK_ isolates had acquired additional lysogenic prophage elements that were associated with enhanced T-cell activation responses.

### Characterization of M1_UK_ strains in experimental skin infection

**Fig 5:**
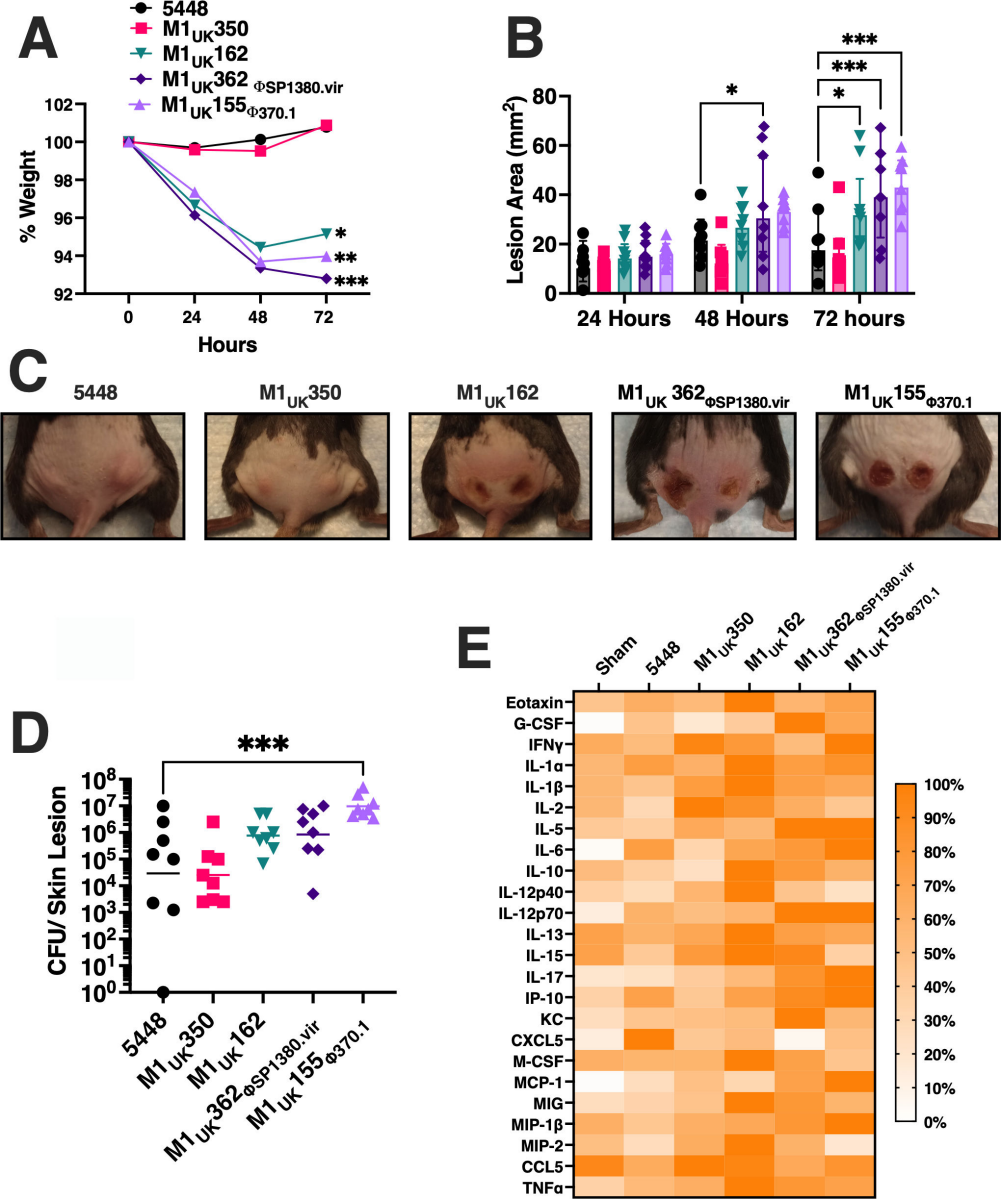
Comparison of *in vivo* infection with the *Streptococcus pyogenes* M1_UK_ sublineage using the skin infection model with B6_HLA_ mice. (**A**) Resulting from the weight tracking daily, it was possible to observe a significant weight decrease starting at 48 h in M1_UK_162, M1_UK_362_ΦSP1380.vir_, and M1_UK_155_Φ370_ groups. Two-way ANOVA (*P < 0.033, **P < 0.002, ***P < 0.0002), each dot represents the average of weight per group (*n* = 4). (**B**) Similarly, the skin lesion areas on the mice were significantly higher in the same groups at 72 h post-infection. Two-way ANOVA (*P < 0.033, ***P < 0.0002)*,* bars represent the geometric mean, while data points represent individual lesions (*n* = 8)*.* (**C**) Representative skin lesions per group after 72 h of skin infection. (**D**) After quantification of recovered bacteria in the skin lesion, only M1_UK_155_Φ370.1_ showed a significant increase in the recovered CFU present in the skin as compared to M1_global_. Kruskal-Wallis test (***P < 0.0002), bars represent the geometric mean, while each data point represents CFU in a single lesion (*n* = 8). (**E**) Cytokine/chemokine profiles evaluated in blood serum after skin infections with GAS strains. A heat map presents normalized data from the highest concentration as 100% per molecule evaluated.

To evaluate virulence characteristics between the different M1 serotype strains, transgenic mice expressing human MHC class II (MHC-II) molecules were challenged using a skin infection model. Superantigens directly bind to both TCRs and MHC-II molecules as host receptors; however, streptococcal superantigens bind with weak affinity to most mouse MHC-II molecules, whereas mice expressing human MHC-II allow for the function of the streptococcal superantigens *in vivo* (11, 17, 18). From these experiments, *S. pyogenes* strains 5448 and M1_UK_350 exhibited similar skin infection characteristics where mice did not lose weight (Fig. 5A), had similar lesion sizes (Fig. 5B and C), and comparable CFUs by 72 h (Fig. 5D). However, *S. pyogenes* M1_UK_162, M1_UK_362_ΦSP1380.vir_, and M1_UK_155_Φ370.1_ each exhibited a significant decrease in weight over the 72 h post-infection compared to the 5448 strain. Moreover, there was an observable increase in the lesion sizes caused by M1_UK_162, M1_UK_362_ΦSP1380.vir_, and M1_UK_155_Φ370_ that were significantly larger at 72 h post-infection (Fig. 5B). Visually, mice infected with *S. pyogenes* 5448 or M1_UK_350 showed less inflamed lesions, characterized by less infiltrates and the absence of dermonecrotic lesions (Fig. 5C). Mice infected with the M1_UK_155_Φ370.1_ also exhibited a significant increase in the bacterial burden per lesion by 72 h (Fig. 5D). Finally, to assess systemic inflammation in response to the subcutaneous skin infection with the M1_global_ or M1_UK_ strains, a cytokine array was used to quantify cytokines and chemokines from serum 72 h post-infection (Fig. 5E). Mice infected with M1_UK_350 showed an increased trend for IFNγ and IL-2, while mice infected with M1_UK_162 displayed elevated trends for eotaxin, IL-12p40, M-CSF, MIG, and TNFα compared to the sham and M1_global_-infected mice. Additionally, infection with the M1_UK_362_ΦSP1380.vir_ strain generated an increased production of G-CSF and KC, and M1_UK_155_Φ370.1_ induced elevated quantities of IL-6, IL-17, IP-10, MCP-1, and MIP-1β. Taken together, we observed an elevated trend for proinflammatory molecules in the infections involving the M1_UK_ strains containing either the *covS* mutation or the additional bacteriophages (Fig. S1).

## DISCUSSION

*S. pyogenes* possesses several toxic and tissue-damaging virulence factors, including cytolytic toxins, proteases, superantigens, and DNases (19), and horizontal gene acquisition of exotoxin-encoding bacteriophages can play an important role in bacterial evolution and the generation of more pathogenic strains (18). Furthermore, hypervirulent strains of *S. pyogenes* can emerge through mutations of the control-of-virulence (Cov) two-component system that dramatically alters global gene regulation with enhanced expression of multiple virulence factors (20, 21). Since the initial report of the emergence of an M1 serotype sublineage termed *S. pyogenes* M1_UK_, there has been a significant increase in invasive cases caused by this *emm1* variant detected in multiple countries worldwide (8, 9, 22–29), including Canada (30, 31). Recently, the national iGAS surveillance program has reported that M1_UK_ has expanded considerably in Canada, increasing from 48.5% in 2022 to 60.3% in 2023 (32). To help understand factors that have contributed to the expansion of *S. pyogenes* M1_UK_, here we aimed to correlate genotype-to-phenotype associations using both *in vitro* and *in vivo* characteristics with a series of contemporary *S. pyogenes* M1_UK_ isolates in comparison with the *S. pyogenes* 5448 M1_global_ strain. Our findings indicated that the M1_UK_ isolates continue to evolve with the acquisition of additional bacteriophage-encoded virulence factors that contribute to exacerbated disease.

One of the key phenotypic differences between the classic circulating M1 strains in comparison with the M1_UK_ sublineage is the overexpression of the superantigen SpeA (8, 9). SpeA has been historically recognized as an important cause of scarlet fever (33) and streptococcal toxic shock syndrome (10, 34), although those disease manifestations do not likely explain the expansion of *S. pyogenes* M1_UK_. Bacterial superantigens function by engaging T-cell receptor β-chains and lateral surfaces of MHC-II molecules to promote a T-cell-dependent inflammatory response (35, 36). However, characterization of superantigen function *in vivo* is challenging, as most mouse MHC-II molecules are not efficiently recognized by bacterial superantigens and thus conventional mice (e.g., C57Bl/6) are relatively insensitive to the effects of these exotoxins (11, 17, 37). To overcome this limitation, multiple studies have evaluated the role of superantigens *in vivo* using “humanized” mice that are transgenic for human MHC-II molecules. From these experiments to date using acute invasive or skin infection models with human MHC-II transgenic mouse models, the collective data suggest that streptococcal superantigens contribute to disease severity, but do not appear to contribute to *S. pyogenes* survival during invasive infection models (37, 38). However, superantigens such as SpeA can promote localized nasopharyngeal infection (11, 17, 18), providing one logical mechanism explaining the increased expansion of the M1_UK_ lineage across multiple countries.

As expected, each of the Canadian M1_UK_ isolates produced enhanced levels of SpeA (Fig. 2B). However, we did not detect any other notable *in vivo* phenotypic differences when comparing *S. pyogenes* 5448 M1_global_ strain with *S. pyogenes* M1_UK_350, suggesting that enhanced SpeA production does not contribute to experimental skin infection. We also evaluated the M1_UK_162 isolate as this strain contained the Leu_238_→Phe_238_ (L238F) mutation within the CovS histidine kinase. CovRS (also referred to as CsrRS [39]) is a well-characterized and pleiotropic two-component regulatory system that may directly or indirectly regulate ~15% of genes in *S. pyogenes* genome (40). Consistent with prior studies in strains containing inactivating mutations within this two-component system (40, 41), M1_UK_162 had a dramatically altered exoprotein profile (Fig. 2A), did not produce detectable levels of the SpeB cysteine protease, but overproduced the SLO toxin (Fig. 2B). Cov mutants also overproduce SpeA (42), and from the Western immunoblot experiments, M1_UK_162 did appear to produce elevated SpeA levels above the other M1_UK_ isolates. As SpeA is relatively resistant to degradation by the SpeB protease (43), the elevated levels of SpeA logically reflect both a depression of *speA* transcription via the *covS* mutation (42) combined with increased transcriptional read-through from the *ssrA* promoter (9). Recently, an Australian M1_UK_ isolate containing a *covS* mutation was characterized to have only subtle differences in virulence factor changes, with no apparent changes in SpeB expression (44). This further adds to the complexity of the CovRS two-component system in *S. pyogenes* and the importance of phenotypic strain characterization for M1 strains.

Strains containing either the ΦSP1380.vir bacteriophage (M1_UK_362) or the Φ370.1 bacteriophage (M1_UK_155), when compared to *S. pyogenes* 5448 or M1_UK_350, each induced more potent human T-cell activation (Fig. 4), which was entirely consistent with the ability of these isolates to encode and produce additional superantigens (Fig. 1A and 2B). Skin infection using these isolates also resulted in larger skin lesions and significant pathophysiological changes in mice following skin infection. Additionally, the M1_UK_162 strain induced similar inflammatory responses to the extra virulence factor strains in the skin infection model (Fig. 5E). However, despite the apparent enhanced levels of SpeA produced from both M1_UK_350 and M1_UK_162 relative to strain 5448, supernatants from these strains did not result in enhanced IL-2 production from human T cells, whereas both isolates that had acquired the addition superantigen-encoded bacteriophages demonstrated did. As superantigens are extremely potent and active T cells in a Vβ-restricted manner, we suspect these results indicate that the SpeA-targeted T cells are fully activated even with the strains that produce less SpeA, whereas SpeC and/or SSA production (from M1_UK_362_ΦSP1380.vir_ or M1_UK_155) will target different T-cell subsets, resulting in the elevated levels of IL-2. The “classic” M1_UK_350 strain showed increased IFN-γ and IL-2 production relative to the other M1_UK_ groups, despite having lower CFUs (Fig. 5D). This may be explained by short-term T-cell exhaustion or anergy induced by strains with enhanced superantigen activity (45), leading to reduced cytokine production by this time point *in vivo*. However, the cytokine and chemokine responses should not be overinterpreted, as these serum samples were collected at the experimental endpoint and therefore likely reflect, at least in part, differences in bacterial burden in addition to effects of bacteriophage-acquired virulence factors or the presence of the *covS* mutation. Concurrent with the lack of enhanced alterations in protease or DNAse activity, cell adhesion, or cytotoxicity from the prophage-containing strains, these data strongly suggest the enhanced skin infection is linked with the production of the additional bacteriophage-encoded superantigens (Fig. 5). Lastly, *S. pyogenes* strains with inactivating *covRS* mutations are considered to be hypervirulent, but also selected against during upper respiratory tract infection (42), yet the skin infections were similar between M1_UK_162, M1_UK_362_ΦSP1380.vir_, and M1_UK_155, suggesting that M1_UK_362_ΦSP1380.vir_ and M1_UK_155_Φ370.1_ may also be hypervirulent in humans, but additionally, may not be selected against during throat colonization.

We did attempt to evaluate these isolates using a nasopharyngeal infection model in the human MHC-II transgenic mice (11); however, M1 serotypes do not cause productive infections in this model for unknown reasons. Nevertheless, these represent acute infection models, whereas *S. pyogenes* can also colonize humans chronically without causing disease (46). Furthermore, superantigens can dampen the host humoral immune response through the suppression of B cells (12, 13). This latter activity provides an additional mechanism by which superantigen acquisition by *S. pyogenes* could potentially contribute to long-term human colonization.

Recent epidemiological screening has now identified M1_UK_ strains containing the Spd1 and SpeC encoding prophage Φ370.1, or the Spd1, SpeC, and SSA encoding prophage ΦSP1380.vir, from multiple regions, including Canada (31), the United States (47), Europe (48, 49), Asia (28), and Australia (9). However, due to limited evidence, there is still no reported correlation between virulence factor acquisition and disease invasiveness in patients. This requires further exploration in future studies. These M1_UK_ strains demonstrated greater immune activation and pathophysiological changes in the host, specifically with the hypervirulent strain with the *covS* mutation, and the strains that have acquired additional virulence factors via bacteriophages. Clear reasons for the widespread expansion of *S. pyogenes* M1_UK_ still remain unclear, but these mechanisms likely involve a key role for SpeA, but also combined with a reduction in mitigation strategies from the COVID-19 pandemic, potentially reflecting diminished immunity to *S. pyogenes*, particularly in children (48). This work used *in vitro* and *in vivo* models to differentiate the functional activity between the M1_global_ strain and four variants of the now circulating, hypervirulent M1_UK_ strains. With these new genomic exchanges and additions driven by the circulating M1_UK_ strains in the population, it is important to continue monitoring the cases generated by these strains in hopes of preventing or controlling epidemic outbreaks.

## MATERIALS AND METHODS

### Bacterial strains, genome comparison, phage induction, and purification

*S. pyogenes* strains used in this study are listed in Table 1 and were routinely cultured on tryptic soy agar (TSA) supplemented with 5% sheep blood (BD Biosciences, USA). For liquid growth, bacteria were grown in Todd Hewitt broth supplemented with 1% (wt/vol) yeast extract (THY) and incubated statically at 37°C. The *S. pyogenes* M1_UK_ isolates were obtained from passive, laboratory-based surveillance systems for invasive GAS in Canada (31), and genome sequences were obtained using Illumina NextSeq technology. The assemblies have been deposited at the National Center for Biotechnology Information (NCBI) (BioSamples SAMN54176055, SAMN54176056, SAMN54176057, SAMN54176058).

The presence of SNPs, deletions, and insertions in the M1_UK_ genomes was done in comparison to the MGAS5005 reference genome (GenBank: NC_007297.2) and was obtained using snippy v4.6.0 (https://github.com/tseemann/snippy). To identify genetic differences in virulence factors, present in each strain, assembled data were processed using BLAST-Virulence Factor Database (http://www.mgc.ac.cn/VFs/); genes were considered detected if reads mapped with greater than 90% coverage and 90% identity. Four M1_UK_ strains were chosen based on genetic differences, including *S. pyogenes* M1_UK_350 as a “classic” M1_UK_ strain, M1_UK_162 that contained a mutation within *covS*, M1_UK_362_ΦSP1380.vir_ that had acquired a lysogenic bacteriophage encoding the *spd1*, *speC*, and *ssa* genes, and M1_UK_155_Φ370.1_ that had acquired a lysogenic bacteriophage containing phage-encoded *spd1* and *speC* genes. For comparison, *S. pyogenes* 5448 was used as a well-studied and representative M1_global_ strain (50).

The virulence genes *speA*, *speC*, *ssa*, *spd1, speB,* and *slo* were PCR amplified from genomic DNA of *S. pyogenes* strains using the MangoTaq DNA polymerase PCR kit (Meridian Bioscience, USA), with primers listed in Table 2. The PCR products were analyzed using 1% agarose gel electrophoresis, with bands visualized through a GelDoc XR+ imaging system (Bio-Rad, USA).

**TABLE 2:** Primers for PCR screening of *S. pyogenes* toxins and bacteriophage elements

Primer	Sequence (5′ to 3′)	Source
*speA*_F	TGTTTCAGGGCCAAATTATGA	(9)
*speA*_R	CATGCACTCCTTTCTGCATT	(9)
*speC*_F	CACACCGGTGAGTACATCTATGGAGG	This study
*speC*_R	CTGATCTAGTCCCTTCATTTGGTGAGTC	This study
*ssa*_F	GATCAAATATTGCTCCAGGTGCGGGC	This study
*ssa*_R	GGGACTAATGTAAGATCCACAGGTCAGC	This study
*spd1*_F	CTGTTGACGCAGCTAGGGTACGAAC	This study
*spd1*_R	GCAACTCACCAGATGAATCAATTCCAACATAC	This study
*speB*-F	TGCTGACGGACGTAACTTCT	(51)
*speB*-R	CCACCAGTACCAAGAGCTGA	(51)
*slo.*tox_F	GCTGGCTAATAAAGGTTTTACCG	(51)
*slo.*tox_R	CGGTAAAACCTTTATTAGCCAGC	(51)
⏀SP1380.vir_F	CTTGAGGCTTGTGGAAGCTGAAGAA	This study
⏀SP1380.vir_R	TCGGATAAGTTCGTACCCTAGCTGC	This study
⏀SP1380.vir_seq_F	CACACCCACGCTTCGTTA	This study
⏀SP1380.vir_seq_R	TGAAGTAGTGGGAGAAGTGA	This study

For the phage induction experiments, bacterial cultures were grown in THY to an OD_600_ of 0.25 and were either induced with subinhibitory concentrations of mitomycin C (Sigma-Aldrich, Germany; 0.2 µg/mL) for phage induction or left untreated. Following three hours of induction at 37˚C*,* phages were harvested by centrifugation (8,000 × *g* for 10 min) from culture supernatants following filtration through 0.22  µm membrane to remove residual bacteria. To eliminate contaminating bacterial DNA, samples were treated with DNase I (Qiagen, Germany; 1 U/µL) for 1.5 h at 37°C. Phage capsids were then digested with proteinase K (Qiagen, Germany; 20 mg/mL; final concentration 0.2 µg/mL) in the presence of 20 mM EDTA for 1.5 h at 56°C to inactivate DNase I. Phage DNA was purified using the DNeasy Blood & Tissue Kit (Qiagen, Germany). The circularized ΦSP1380.vir was detected using inverse PCR with primers (Table 2) flanking the attachment site of the ΦSP1380.vir prophage, using the KAPA HiFi PCR kit (Roche, Switzerland) according to the manufacturer’s instructions. To confirm the absence of contaminating genomic DNA, samples were tested with *slo* PCR primers using the MangoTaq DNA Polymerase PCR kit (Meridian Bioscience, BIO-21083; USA). PCR products were analyzed on 1% agarose gels and visualized. PCR products detecting ΦSP1380.vir were sequenced (Genetic Research Services, University of Queensland, Brisbane, Australia) with the primers listed previously.

### Comparison of secreted virulence factors from M1_UK_ isolates

To characterize exotoxin expression from the different isolates, *S. pyogenes* strains were cultured to late-exponential phase in THY broth, and supernatants were filtered through a 0.45 μm filter, precipitated with 6% trichloroacetic acid, washed with acetone, and resuspended in 8M urea. Proteins were then subjected to SDS-PAGE, and gels were stained using Ready Blue stain (Milipore, USA).

Identification of the exoproteins SpeA, SpeC, SSA, Spd1, SpeB, and SLO in the supernatants was performed by Western blotting as previously described, using rabbit or mouse antisera (18). Briefly, samples were separated by 12% SDS-PAGE and transferred onto methanol-activated PVDF membranes (Merck, IPFL00010; Germany) using a wet transfer system (Bio-Rad, USA). Membranes were blocked in Intercept Blocking Buffer (LI-COR, 927-70003; USA) for 1  h at room temperature, followed by overnight incubation at 4°C with primary antibodies targeting SpeA (PAI111, Toxin Technology, USA; 1:1,000 dilution), SpeC (PCI333, Toxin Technology, USA; 1:1,000 dilution), SSA (Mimotopes, Australia; 1:500 dilution), and SpeB (PBI222; Toxin Technology, USA; 1:1,000 dilution). Spd1 and SLO were detected using murine antibodies at 1:1,000 and 1:2,000 dilutions, respectively. Fluorescent secondary antibodies (DyLight 800 anti-mouse or anti-rabbit IgG) were applied for 1  h at room temperature, and membranes were imaged using an Odyssey Imaging System (LI-COR biosciences, USA).

### SpeB proteolysis activity

To evaluate proteolytic activity, brain heart infusion (BHI) media (Difco, USA) was dialyzed using 12–14 kDa dialysis membrane (Spectrum Spectra/Por) in water for 18 h at 4°C. Dialyzed BHI was then used to make 1.5% (wt/vol) agar plates supplemented with 3% (wt/vol) skim milk powder, and 10 μL of each *S. pyogenes* isolate, cultivated to an OD_600_ of 0.1, was plated in quadruplicate and incubated at 37°C for 24 h. Zones of clearance indicating casein hydrolysis were measured and imaged (11).

### Host cell adhesion assays

For cell adhesion assays, Detroit-562 pharyngeal cells (ATCC CCL-138) were seeded at a concentration of 200,000 cells per well in 24-well tissue culture plates (Fisherbrand, USA) in complete MEM media (cMEM) (10% Glutamax, 10% FBS; GIBCO). *S. pyogenes* strains were grown to early stationary phase (OD_600_ ~ 0.2–0.4), and the desired volume of bacteria was washed three times with PBS. Bacterial concentrations were adjusted to achieve a multiplicity of infection of 10, and plates were incubated for 2 h at 37°C in 5% CO_2_. Following incubation, wells were washed three times to remove unbound bacteria and incubated with 500 μL of PBS containing 0.1% (vol/vol) Triton X-100 for 5 min at 37°C to lyse the cell layer. Supernatants were collected and serially diluted before being plated on TSA supplemented with 5% sheep blood and incubated overnight to quantify the recovered bacteria (52).

### Lactate dehydrogenase assay

The quantification of extracellular lactate dehydrogenase released from pharyngeal cells was used to determine the bacterial supernatant cytotoxicity. Bacterial supernatants were obtained from cultures grown to an OD_600_ of 0.6–0.8. Detroit-562 cells were seeded at a concentration of 30,000 cells per well in a 96-well plate in cMEM. Cells were washed three times with PBS and incubated in a final volume of 180 μL of MEM without FBS and 20 μL of bacterial supernatant (10^−1^ dilution factor was used). As a positive control, Triton X-100 was added to lyse all cells, and media-only wells were used as a background control. After 3 h of incubation, plates were centrifuged at 400 × *g* for 5 min, and supernatants were collected. Samples were processed according to the manufacturer’s instructions (Sigma-Aldrich, #MAK066, USA), and the absorbance was measured using an absorbance of 450 nm (Biotek Synergy H4, USA). Results were calculated and presented as the percentage of cell damage generated by the M1_global_ and M1_UK_ strain’s supernatants (53).

### Degradation of DNA and NETs by M1_UK_ supernatants

To assess secreted DNase activity from the M1_UK_ isolates, PCR products were tested for degradation by bacterial supernatants. PCR products generated using the *speC* primers were mixed with 5 μL of raw supernatant, supernatant diluted 1:100, and 1:1,000. The reaction was incubated for 2 h at 37°C and stopped by the addition of 1M EDTA. Products were analyzed and imaged using 1% agarose gel electrophoresis (15).

To evaluate extracellular DNase activity from the M1_UK_ isolates, human neutrophils from healthy human donors were isolated using a Ficoll-Paque density gradient protocol, seeded at 2 × 10^5^ cells in a chambered cell culture slide (MatTek, USA), and incubated in RPMI 1640 supplemented with 10% Glutamax, 100 μg/mL of streptomycin (Gibco), and 100 U/mL of penicillin (Gibco) (18). To induce the formation of NETosis, cells were stimulated with 100 ng/mL of Phorbol 12-Myristate 13-Acetate (PMA) for 4 h. After induction, cultures were washed five times with PBS and re-cultured with RPMI containing prefiltered bacterial supernatant (100-fold dilution) for 2 h. Next, cells were washed and fixed overnight with Zinc Formalin Fixative (Sigma, USA). Samples were washed five times and permeabilized with PBS containing 0.1% (vol/vol) Triton X-100 for 30 min at 37°C. After three washes, samples were incubated with 5 μg/mL DAPI for 30 min and mounted using Prologold mounting media (Thermo Fisher, USA). Images were obtained using an Olympus BX61 fluorescence microscope. To quantify degradation of NETs from the acquired images, relative fluorescence intensity was measured using Image J (54). Briefly, each image (2,048 × 1,536 pixels) was analyzed on the single-colored images stained with DAPI, and the background was normalized at 0.35% of saturated pixels, and the mean of the fluorescence was collected for each sample. A total of eight images were analyzed per sample.

### Human PBMC activation assays

To assess the ability of the M1_UK_ strains to cause immune cell activation, IL-2 production was measured from PBMCs stimulated with prefiltered bacterial supernatants from the late-exponential phase. Venous blood was taken from healthy donors in accordance with a human subject protocol approved by the London Health Sciences Center (LHSC) Research Ethics Boards at the University of Western Ontario, London, Ontario, Canada (Protocol 110859). Volunteers were recruited by a passive advertising campaign within the Department of Microbiology and Immunology at the University of Western Ontario, and written informed consent was given by each volunteer before each sample was taken. Following sampling, blood was fully anonymized, and no information regarding the identity of the donor, including sex and age, was retained. PBMCs were obtained from three healthy donors using the Ficoll-Paque density gradient protocol and seeded at a final concentration of 2 × 10^6^ cells per well in a 96-well plate (11). Cells were treated with serially diluted supernatants from the M1_global_ or M1_UK_ strains (10^−1^ to 10^−7^ dilution) in triplicate and incubated for 18 h at 37°C with 5% CO_2_. Cell supernatants were collected to quantify the concentration of human IL-2 using ELISA as per the manufacturer’s instructions (Invitrogen) (11).

### Mouse skin infection model

To evaluate the pathophysiological differences caused by the M1_global_ and M1_UK_ strains during the course of an infection, a skin infection model using B6_HLA_ mice (transgenic for the HLA-DR4/DQ8 genes) was used (16, 52)*.* Experiments were carried out following the Canadian Council on Animal Care Guide to the Care and Use of Experimental Animals and the animal protocol, approved by the Animal Use Subcommittee at the University of Western Ontario (Protocol 2024-105). Mice between 8 and 12 weeks old were shaved and treated with hair removal cream on the lower back one day before infection with *S. pyogenes*. Mice were anesthetized and then injected subcutaneously with 5 × 10^6^ CFU of bacteria in each flank. Lesion sizes and mouse weights were measured daily over 72 h, after which time mice were sacrificed, and skin lesions were excised, homogenized, and plated to evaluate bacterial burden. Additionally, blood was obtained using intracardiac collection in heparinized needles and centrifuged to collect sera. Samples were stored at −20°C for future analysis (16, 52).

### Multiplex cytokines and interleukins analysis

The immunological response from the B6_HLA_ mice sera after skin infection with the *S. pyogenes* strains was evaluated utilizing the Mouse Cytokine/Chemokine 32-Plex Discovery Assay (Eve Technologies, Canada). Uninfected B6_HLA_ mice were used as background control (Sham). A total of 24 cytokine/chemokine molecules were detected in the serum, and data were analyzed and plotted (Fig. S1). Statistical comparison of the groups was performed against the 5448 results. There were no detectable values for the rest of the molecules evaluated in the assay. The data were presented as a heat map where each cytokine quantity was normalized to 100%, which represents the highest concentration detected from any condition.

### Statistical analysis

All statistical analysis presented in this study was conducted using GraphPad Prism 9.0.

## SUPPLEMENTAL MATERIAL

Supplemental material iai.00503-25-s0001.pdf Tables S1 and S2; Fig. S1.

ASM does not own the copyrights to Supplemental Material that may be linked to, or accessed through, an article. The authors have granted ASM a non-exclusive, world-wide license to publish the Supplemental Material files. Please contact the corresponding author directly for reuse.
