# Supplementary material for: Acquisition of toxin-encoding lysogenic bacteriophage elements enhances the virulence of pandemic Streptococcus pyogenes M1UK
Source: Infect Immun. 2026 Feb 9;94(3):e00503-25. doi: 10.1128/iai.00503-25 (PMC12974143; doi:10.1128/iai.00503-25)
Supplement: Supplemental material — Tables S1 and S2; Fig. S1. [file iai.00503-25-s0001.pdf]

Supplemental material

Acquisition of toxin-encoding lysogenic bacteriophage elements enhances virulence of pandemic *Streptococcus pyogenes* $M1_{UK}$

Juan Manuel Diaz[1], Jasmine Wells[2], Amanda C. Marple[1], Blake A. Shannon[1], Aanchal Rishi[1], Irene Martin[3], Alison McGeer[4,5], Matthew A. Croxen[6,7,8,9], Gregory J. Tyrrell[6,7], Mark J. Walker[2], Stephan Brouwer[2], John K. McCormick[1,*].

[1] Department of Microbiology and Immunology, University of Western Ontario, London, Ontario, Canada

[2] Australian Infectious Diseases Research Centre and Institute for Molecular Biosciences, The University of Queensland, St. Lucia, QLD, Australia.

[3] National Microbiology Laboratory, Public Health Agency of Canada, Winnipeg, Manitoba, Canada

[4] Department of Microbiology, Mount Sinai Hospital, Toronto, Ontario, Canada

[5] Department of Laboratory Medicine and Pathobiology, University of Toronto, Toronto, Ontario, Canada

[6] Department of Laboratory Medicine and Pathology, Faculty of Medicine and Dentistry, University of Alberta, Edmonton, Alberta, Canada

[7] Public Health Laboratory -Alberta Precision Laboratories, Edmonton, Alberta, Canada

[8] Li Ka Shing Institute of Virology, University of Alberta, Edmonton, Alberta, Canada

[9] Women and Children's Health Research Institute, University of Alberta, Edmonton, Alberta, Canada

Table S1. Identification of the 27 SNPs in the *Streptococcus pyogenes* M1$_{UK}$ and the CovS mutation present in the strains used in this study.

Position	Locus Tag	Gene	M1$_{UK}$ Strains			
			M1$_{UK}$350	M1$_{UK}$162	M1$_{UK}$362	M1$_{UK}$155
115646	M5005_RS00740	Transcriptional regulator, RofA	+	+	+	+
116162	M5005_RS00740	Transcriptional regulator, RofA	+	+	+	+
116163	M5005_RS00740	Transcriptional regulator, RofA	+	+	+	+
250832	M5005_RS01370	Fe-S cluster assembly protein, SufD	+	+	+	+
513254	M5005_RS02715	RpiB/LacA/LacB family sugar-phosphate isomerase	+	+	+	+
528360	Intergenic		+	+	+	+
563631	M5005_RS02915	CPBP family intramembrane metalloprotease	+	+	+	+
613633	M5005_RS03130	LTA synthase family protein	+	+	+	+
626494	M5005_RS03195	class I SAM-dependent rRNA methyltransferase	+	+	+	+
661707	M5005_RS03355	tRNA (guanosine(37)-N1)-methyltransferase, TrmD	+	+	+	+
730823	M5005_RS03700	single-stranded-DNA-specific exonuclease, RecJ	+	+	+	+
784467	M5005_RS03935	YeiH family protein	+	+	+	+
819098	M5005_RS04145	UDP-N-acetylmuramate dehydrogenase, MurB	+	+	+	+
923079	M5005_RS04650	NADH-dependent flavin oxidoreductase	+	+	+	+
942633	M5005_RS04740	phosphate ABC transporter ATP-binding protein, PstB	+	+	+	+
983438	Intergenic		+	+	+	+
1082253	M5005_RS05485	methionine adenosyltransferase, MetK	+	+	+	+
1238124	M5005_RS06325	peptide-methionine (R)-S-oxide reductase MsrB	+	+	+	+
1238673	M5005_RS06330	TlpA disulfide reductase family protein	+	+	+	+
1251193	M5005_RS06380	AAA family ATPase	+	+	+	+
1373176	M5005_RS06950	PTS sugar transporter subunit IIB	+	+	+	+
1407497	M5005_RS07140	phage portal protein	+	+	+	+
1446116	M5005_RS07380	3-oxoacyl-[acyl-carrier-protein] reductase, FabG	+	+	+	+
1535209	Intergenic		+	+	+	+
1702540	M5005_RS08565	glycerol dehydrogenase	+	+	+	+
1734749	M5005_RS08715	glutamate formimidoyltransferase, FtcD	+	+	+	+
1828734	M5005_RS09140	YitT family protein	+	+	+	+
**293229**[ǂ]	M5005_RS01585	two-component system sensor histidine kinase CovS	-	+	-	-

*Note: Alignments were referenced from NCBI Accession NC_007297. **SNP present "+". SNP absent "-"**.
"ǂ" Variant call: stop_lost c.713A>T p.Ter238Leuext*?.

Table S2. Other SNPs, deletions, and mutations identified in the *Streptococcus pyogenes* M1UK strains used in this study.

***Streptococcus pyogenes* M1UK 350**			
**Position**	**Locus Tag**	**Gene**	**Variant Description**
33566	M5005_RS00245	ribose-phosphate diphosphokinase	missense_variant c.954G>A p.Met318Ile
76208	M5005_RS00475	adenylate kinase	disruptive_inframe_deletion c.453_455delAGA p.Glu151del
78299	M5005_RS00495	DNA-directed RNA polymerase subunit alpha	missense_variant c.590T>C p.Met197Thr
111953	M5005_RS00715	DUF4479 and tRNA-binding domain-containing protein	synonymous_variant c.408G>A p.Ala136Ala
128773	M5005_RS09265	LysR family transcriptional regulator	missense_variant c.628A>G p.Ser210Gly
167489	Intergenic		Snp. C>T
291797	M5005_RS01575	DUF177 domain-containing protein	missense_variant c.136C>T p.Arg46Cys
313626	M5005_RS01685	glutamate racemase, *racE*	missense_variant c.482C>T p.Ala161Val
338210	M5005_RS01825	DNA polymerase III subunit delta'	missense_variant c.524A>G p.Glu175Gly
343279	Intergenic		Del. TA>T
481647	Intergenic		Ins. T>TAGAAA
547754	M5005_RS02870	phosphopyruvate hydratase, *eno*	missense_variant c.159_165delCGTGTCAinsTGGTGAC p.ValSer54GlyAsp
559838	M5005_RS02895	TOMM family cytolysin streptolysin S, *sagA*	synonymous_variant c.102A>G p.Ser34Ser
592949	M5005_RS03055	ATP-dependent nuclease subunit B, *rexB*	missense_variant c.424A>C p.Lys142Gln
603488	M5005_RS03085	RNA polymerase sigma factor RpoD, *rpoD*	missense_variant c.406G>A p.Ala136Thr
629986	Intergenic		Snp. G>A
687224	M5005_RS03480	phosphomevalonate kinase	missense_variant c.799C>A p.Leu267Ile
712345	M5005_RS03605	dihydroorotase	missense_variant c.1045C>G p.Leu349Val
768350	M5005_RS03860	phosphoglucosamine mutase, *glmM*	missense_variant c.939C>A p.Asp313Glu
779899	M5005_RS09825	nucleoside-diphosphate kinase	missense_variant c.113T>C p.Leu38Pro
782255	Intergenic		Snp. A>G
791442	M5005_RS03970	extracellular solute-binding protein	missense_variant c.1034T>A p.Val345Glu
802016	Intergenic		Snp. T>G
850245	M5005_RS04300	nucleobase:cation symporter-2 family protein	missense_variant c.305A>T p.Lys102Met
937373	M5005_RS04720	HAMP domain-containing sensor histidine kinase	synonymous_variant c.393A>G p.Gly131Gly
942163	M5005_RS04735	phosphate signaling complex protein PhoU, *phoU*	missense_variant c.11C>T p.Thr4Met
957886	Intergenic		Snp. T>C
959346	Intergenic		Snp. C>T
1046996	M5005_RS05320	D-alanine--poly(phosphoribitol) ligase subunit DltA, *dltA*	missense_variant c.193A>G p.Asn65Asp
1064592	M5005_RS05405	pseudouridine synthase	synonymous_variant c.705G>A p.Leu235Leu
1121024	M5005_RS05665	hypothetical protein	missense_variant c.146A>G p.Lys49Arg
1147752	M5005_RS05790	glucosaminidase domain-containing protein	synonymous_variant c.1152T>C p.Ile384Ile
1205792	M5005_RS06170	cell division protein FtsZ, *ftsZ*	missense_variant c.197T>C p.Leu66Ser
1212101	M5005_RS06200	translational GTPase TypA, *typA*	missense_variant c.1661C>T p.Ala554Val
1219790	M5005_RS06240	LacI family DNA-binding transcriptional regulator	synonymous_variant c.465A>G p.Gly155Gly
1233554	M5005_RS06310	MmcQ/YjbR family DNA-binding protein	missense_variant c.928T>C p.Cys310Arg
1242693	M5005_RS06355	type I-C CRISPR-associated protein Cas7/Csd2, *cas7c*	missense_variant c.368C>T p.Ser123Phe
1294671	Intergenic		Del. CT>C
1350795	Intergenic		Del. GT>G
1391334	M5005_RS07050	gp58-like family protein	synonymous_variant c.252T>G p.Ala84Ala
1458349	M5005_RS07445	histidine phosphatase family protein	missense_variant c.563T>C p.Leu188Pro
1467405	M5005_RS07495	universal stress protein	missense_variant c.86T>C p.Val29Ala
1488191	M5005_RS07570	heme-binding protein Shr, *shr*	synonymous_variant c.486T>C p.Gly162Gly
1494928	Intergenic		Del. AT>A
1568351	M5005_RS07960	DNA-directed RNA polymerase subunit delta	synonymous_variant c.564A>G p.Glu188Glu

1624722	M5005_RS08250	NADPH-dependent FMN reductase	synonymous_variant c.123T>C p.Asp41Asp
1630160	Intergenic		Del. CT>C
1636851	M5005_RS08295	pullulanase	synonymous_variant c.2943T>C p.Ala981Ala
1681273	M5005_RS08470	complement-mediated lysis inhibitor Sic, *sic*	disruptive_inframe_deletion c.666_674delCTTTGATGG p.Phe223_Gly225del
1732470	M5005_RS08710	urocanate hydratase	synonymous_variant c.21C>T p.Thr7Thr
1809755	M5005_RS09055	L-serine ammonia-lyase, iron-sulfur-dependent, subunit alpha, *sdaAA*	synonymous_variant c.330T>C p.Val110Val

Streptococcus pyogenes M1$_{UK}$ 162

Position	Locus Tag	Gene	Variant Description
33566	M5005_RS00245	ribose-phosphate diphosphokinase	missense_variant c.954G>A p.Met318Ile
76208	M5005_RS00475	adenylate kinase	disruptive_inframe_deletion c.453_455delAGA p.Glu151del
78299	M5005_RS00495	DNA-directed RNA polymerase subunit alpha	missense_variant c.590T>C p.Met197Thr
111953	M5005_RS00715	DUF4479 and tRNA-binding domain-containing protein	synonymous_variant c.408G>A p.Ala136Ala
144839	M5005_RS00885	toxic anion resistance protein	frameshift_variant c.279delA p.Lys93fs
167489			snp
197209	M5005_RS09290	ISAs1-like element IS1548 family transposase	synonymous_variant c.1120T>C p.Leu374Leu
261865	M5005_RS01410	ABC transporter permease	synonymous_variant c.780A>G p.Gly260Gly
272606	M5005_RS10005	hypothetical protein	missense_variant c.14T>C p.Val5Ala
291797	M5005_RS01575	DUF177 domain-containing protein	missense_variant c.136C>T p.Arg46Cys
312317	M5005_RS01675	Bax inhibitor-1/YccA family protein	synonymous_variant c.460C>T p.Leu154Leu
317937	Intergenic		Del. GA>G
338210	M5005_RS01825	DNA polymerase III subunit delta'	missense_variant c.524A>G p.Glu175Gly
343279	Intergenic		Del. TA>T
397788	M5005_RS02150	hypothetical protein	missense_variant c.25A>T p.Ile9Leu
419496	M5005_RS02260	threonine--tRNA ligase, *thrS*	missense_variant c.185C>T p.Pro62Leu
504884	Intergenic		Del. CT>C
547754	M5005_RS02870	phosphopyruvate hydratase, *eno*	missense_variant c.159_165delCGTGTCAinsTGGTGAC p.ValSer54GlyAsp
559838	M5005_RS02895	TOMM family cytolysin streptolysin S, *sagA*	synonymous_variant c.102A>G p.Ser34Ser
592949	M5005_RS03055	ATP-dependent nuclease subunit B, *rexB*	missense_variant c.424A>C p.Lys142Gln
603488	M5005_RS03085	RNA polymerase sigma factor RpoD, *rpoD*	missense_variant c.406G>A p.Ala136Thr
638948	M5005_RS03260	LysR family transcriptional regulator	synonymous_variant c.184C>T p.Leu62Leu
665299	M5005_RS03370	2-dehydropantoate 2-reductase	synonymous_variant c.750T>C p.Ala250Ala
687224	M5005_RS03480	phosphomevalonate kinase	missense_variant c.799C>A p.Leu267Ile
768350	M5005_RS03860	phosphoglucosamine mutase, *glmM*	missense_variant c.939C>A p.Asp313Glu
779899	M5005_RS09825	nucleoside-diphosphate kinase	missense_variant c.113T>C p.Leu38Pro
782255	Intergenic		Snp. A>G
782340	Intergenic		Del. GAACAA>G
791442	M5005_RS03970	extracellular solute-binding protein	missense_variant c.1034T>A p.Val345Glu
813028	M5005_RS04110	D-alanyl-D-alanine carboxypeptidase PBP3, *pbp3*	missense_variant c.532G>A p.Ala178Thr
828498	Intergenic		Del. GA>G
850245	M5005_RS04300	nucleobase:cation symporter-2 family protein	missense_variant c.305A>T p.Lys102Met
866110	M5005_RS04375	DNA gyrase subunit A, *gyrA*	synonymous_variant c.711T>A p.Ser237Ser
882013	M5005_RS04455	methylenetetrahydrofolate--tRNA-(uracil(54)- C(5))-methyltransferase (FADH(2)-oxidizing) TrmFO, *trmFO*	synonymous_variant c.579C>T p.Tyr193Tyr
937373	M5005_RS04720	HAMP domain-containing sensor histidine kinase	synonymous_variant c.393A>G p.Gly131Gly
942163	M5005_RS04735	phosphate signaling complex protein PhoU, *phoU*	missense_variant c.11C>T p.Thr4Met
957886	Intergenic		Snp. T>C

959346	Intergenic		Snp. C>T
1046996	M5005_RS05320	D-alanine--poly(phosphoribitol) ligase subunit DltA, *dltA*	missense_variant c.193A>G p.Asn65Asp
1064592	M5005_RS05405	pseudouridine synthase	synonymous_variant c.705G>A p.Leu235Leu
1121024	M5005_RS05665	hypothetical protein	missense_variant c.146A>G p.Lys49Arg
1147752	M5005_RS05790	glucosaminidase domain-containing protein	synonymous_variant c.1152T>C p.Ile384Ile
1212101	M5005_RS06200	translational GTPase TypA, *typA*	missense_variant c.1661C>T p.Ala554Val
1219790	M5005_RS06240	LacI family DNA-binding transcriptional regulator	synonymous_variant c.465A>G p.Gly155Gly
1233554	M5005_RS06310	MmcQ/YjbR family DNA-binding protein	missense_variant c.928T>C p.Cys310Arg
1265144	M5005_RS06450	YesL family protein	missense_variant c.459G>A p.Met153Ile
1294671	Intergenic		Del. CT>C
1391334	M5005_RS07050	gp58-like family protein	synonymous_variant c.252T>G p.Ala84Ala
1458349	M5005_RS07445	histidine phosphatase family protein	missense_variant c.563T>C p.Leu188Pro
1467405	M5005_RS07495	universal stress protein	missense_variant c.86T>C p.Val29Ala
1488191	M5005_RS07570	heme-binding protein Shr, *shr*	synonymous_variant c.486T>C p.Gly162Gly
1547208	M5005_RS07845	uridine phosphorylase, *udp*	synonymous_variant c.409T>C p.Leu137Leu
1568351	M5005_RS07960	DNA-directed RNA polymerase subunit delta, *rpoE*	synonymous_variant c.564A>G p.Glu188Glu
1624722	M5005_RS08250	NADPH-dependent FMN reductase	synonymous_variant c.123T>C p.Asp41Asp
1630160	Intergenic		Del. CT>C
1636851	M5005_RS08295	pullulanase	synonymous_variant c.2943T>C p.Ala981Ala
1681273	M5005_RS08470	complement-mediated lysis inhibitor Sic, *sic*	disruptive_inframe_deletion c.666_674delCTTTGATGG p.Phe223_Gly225del
1681380	M5005_RS08470	complement-mediated lysis inhibitor Sic, *sic*	missense_variant c.568G>A p.Gly190Arg
1683446	M5005_RS08475	M protein, *emm*	missense_variant c.145G>A p.Glu49Lys
1732470	M5005_RS08710	urocanate hydratase	synonymous_variant c.21C>T p.Thr7Thr
1809755	M5005_RS09055	L-serine ammonia-lyase, iron-sulfur-dependent, subunit alpha, *sdaAA*	synonymous_variant c.330T>C p.Val110Val
1826928	Intergenic		Ins. A>ATTAATCAAAAAATTCATTTGGGTCTTATTT

Streptococcus pyogenes M1$_{UK}$ 362

Position	Locus Tag	Gene	Variant Description
33566	M5005_RS00245	ribose-phosphate diphosphokinase	missense_variant c.954G>A p.Met318Ile
76208	M5005_RS00475	adenylate kinase	disruptive_inframe_deletion c.453_455delAGA p.Glu151del
78299	M5005_RS00495	DNA-directed RNA polymerase subunit alpha	missense_variant c.590T>C p.Met197Thr
106304	M5005_RS00680	class I SAM-dependent methyltransferase	missense_variant c.511C>A p.Pro171Thr
111953	M5005_RS00715	DUF4479 and tRNA-binding domain-containing protein	synonymous_variant c.408G>A p.Ala136Ala
129606	M5005_RS00810	acetyl-CoA C-acetyltransferase	frameshift_variant c.89dupT p.Leu30fs
155552	Intergenic		Snp. T>C
167489	Intergenic		Snp. C>T
182627	Intergenic		Snp. A>G
291797	M5005_RS01575	DUF177 domain-containing protein	missense_variant c.136C>T p.Arg46Cys
338210	M5005_RS01825	DNA polymerase III subunit delta'	missense_variant c.524A>G p.Glu175Gly
343279	Intergenic		Del. TA>T
445837	M5005_RS02370	MFS transporter	frameshift_variant c.20delA p.Asn7fs
456713	M5005_RS10035	histidyl-tRNA synthetase	missense_variant c.389T>A p.Val130Asp
547754	M5005_RS02870	phosphopyruvate hydratase, *eno*	missense_variant c.159_165delCGTGTCAinsTGGTGAC p.ValSer54GlyAsp
559838	M5005_RS02895	TOMM family cytolysin streptolysin S, *sagA*	synonymous_variant c.102A>G p.Ser34Ser
592949	M5005_RS03055	ATP-dependent nuclease subunit B, *rexB*	missense_variant c.424A>C p.Lys142Gln
603488	M5005_RS03085	RNA polymerase sigma factor RpoD, *rpoD*	missense_variant c.406G>A p.Ala136Thr

629986	Intergenic		Snp. G>A
687224	M5005_RS03480	phosphomevalonate kinase	missense_variant c.799C>A p.Leu267Ile
712345	M5005_RS03605	dihydroorotase	missense_variant c.1045C>G p.Leu349Val
768350	M5005_RS03860	phosphoglucosamine mutase, *glmM*	missense_variant c.939C>A p.Asp313Glu
779899	M5005_RS09825	nucleoside-diphosphate kinase	missense_variant c.113T>C p.Leu38Pro
782255	Intergenic		Snp. A>G
782340	Intergenic		Del. GAACAA>G
791442	M5005_RS03970	extracellular solute-binding protein	missense_variant c.1034T>A p.Val345Glu
850245	M5005_RS04300	nucleobase:cation symporter-2 family protein	missense_variant c.305A>T p.Lys102Met
868639	M5005_RS04380	sortase	missense_variant c.734A>T p.Asn245Ile
889745	Intergenic		Snp. C>T
914604	Intergenic		Snp. A>T
937373	M5005_RS04720	HAMP domain-containing sensor histidine kinase	synonymous_variant c.393A>G p.Gly131Gly
942163	M5005_RS04735	phosphate signaling complex protein PhoU, *phoU*	missense_variant c.11C>T p.Thr4Met
957886	Intergenic		Snp. T>C
959346	Intergenic		Snp. C>T
1009159	M5005_RS05110	N-6 DNA methylase	synonymous_variant c.546A>G p.Arg182Arg
1022000	Intergenic		Snp. A>C
1024866	M5005_RS05230	glycogen/starch/alpha-glucan family phosphorylase, *glpP*	missense_variant c.341C>G p.Ala114Gly
1025877	M5005_RS05235	4-alpha-glucanotransferase, *malQ*	synonymous_variant c.858C>T p.Phe286Phe
1046996	M5005_RS05320	D-alanine--poly(phosphoribitol) ligase subunit DltA, *dtlA*	missense_variant c.193A>G p.Asn65Asp
1064592	M5005_RS05405	pseudouridine synthase	synonymous_variant c.705G>A p.Leu235Leu
1121024	M5005_RS05665	hypothetical protein	missense_variant c.146A>G p.Lys49Arg
1147752	M5005_RS05790	glucosaminidase domain-containing protein	synonymous_variant c.1152T>C p.Ile384Ile
1150752	M5005_RS05820	gp58-like family protein	missense_variant c.1708C>G p.His570Asp
1192865	Intergenic		Del. CATAGAACAGATT>C
1205792	M5005_RS06170	cell division protein FtsZ, *ftsZ*	missense_variant c.197T>C p.Leu66Ser
1212101	M5005_RS06200	translational GTPase TypA, *typA*	missense_variant c.1661C>T p.Ala554Val
1219790	M5005_RS06240	LacI family DNA-binding transcriptional regulator	synonymous_variant c.465A>G p.Gly155Gly
1233554	M5005_RS06310	MmcQ/YjbR family DNA-binding protein	missense_variant c.928T>C p.Cys310Arg
1458349	M5005_RS07445	histidine phosphatase family protein	missense_variant c.563T>C p.Leu188Pro
1467405	M5005_RS07495	universal stress protein	missense_variant c.86T>C p.Val29Ala
1482353	M5005_RS07555	iron ABC transporter permease	synonymous_variant c.555C>T p.Pro185Pro
1488191	M5005_RS07570	heme-binding protein Shr, *shr*	synonymous_variant c.486T>C p.Gly162Gly
1568351	M5005_RS07960	DNA-directed RNA polymerase subunit delta, *rpoE*	synonymous_variant c.564A>G p.Glu188Glu
1602942	Intergenic		Snp. A>T
1624722	M5005_RS08250	NADPH-dependent FMN reductase	synonymous_variant c.123T>C p.Asp41Asp
1630160	Intergenic		Del. CT>C
1636851	M5005_RS08295	pullulanase	synonymous_variant c.2943T>C p.Ala981Ala
1681273	M5005_RS08470	complement-mediated lysis inhibitor Sic, *sic*	disruptive_inframe_deletion c.666_674delCTTTGATGG p.Phe223_Gly225del
1683454	M5005_RS08475	M protein, *emm*	missense_variant c.137A>G p.Asn46Ser
1732470	M5005_RS08710	urocanate hydratase	synonymous_variant c.21C>T p.Thr7Thr
1763775	M5005_RS08820	membrane protein	frameshift_variant c.73_74dupAC p.Thr26fs
1807143	Intergenic		Snp. T>A
1809755	M5005_RS09055	L-serine ammonia-lyase, iron-sulfur-dependent, subunit alpha, *sdaAA*	synonymous_variant c.330T>C p.Val110Val
1811375	Intergenic		Snp. A>T
1811394	Intergenic		Del. AT>A

Streptococcus pyogenes M1UK 155			
**Position**	**Locus Tag**	**Gene**	**Variant Description**
33566	M5005_RS00245	ribose-phosphate diphosphokinase	missense_variant c.954G>A p.Met318Ile
53195	Intergenic		Del. TA>T
67817	M5005_RS00395	50S ribosomal protein L22, *rplV*	synonymous_variant c.111C>T p.Ile37Ile
76208	M5005_RS00475	adenylate kinase	disruptive_inframe_deletion c.453_455delAGA p.Glu151del
78299	M5005_RS00495	DNA-directed RNA polymerase subunit alpha	missense_variant c.590T>C p.Met197Thr
107957	M5005_RS00685	acetate kinase	synonymous_variant c.1152A>G p.Glu384Glu
111953	M5005_RS00715	DUF4479 and tRNA-binding domain-containing protein	synonymous_variant c.408G>A p.Ala136Ala
143652	M5005_RS00880	V-type ATP synthase subunit D	synonymous_variant c.543G>A p.Leu181Leu
167489	Intergenic		Snp. C>T
192150	Intergenic		Del. TAAAAAACCACTATGATGAGAATATGCTTGCATTGACAC ATGTTTTATGTCTG>T
291797	M5005_RS01575	DUF177 domain-containing protein	missense_variant c.136C>T p.Arg46Cys
310333	Intergenic		Snp. G>A
338210	M5005_RS01825	DNA polymerase III subunit delta'	missense_variant c.524A>G p.Glu175Gly
343279	Intergenic		Del. TA>T
376123	M5005_RS02020	DNA translocase FtsK	missense_variant c.266T>C p.Met89Thr
389548	M5005_RS02090	uracil-DNA glycosylase family protein	missense_variant c.8A>G p.Asp3Gly
396830	M5005_RS02145	hypothetical protein	missense_variant c.155C>T p.Thr52Ile
409910	M5005_RS02210	pyroglutamyl-peptidase I, *pcp*	synonymous_variant c.213C>T p.Gly71Gly
452758	M5005_RS02420	Fic family protein	disruptive_inframe_deletion c.161_163delCTT p.Ser54del
487718	M5005_RS02615	oligoendopeptidase F, *pepF*	missense_variant c.1688T>C p.Met563Thr
547754	M5005_RS02870	phosphopyruvate hydratase, *eno*	missense_variant c.159_165delCGTGTCAinsTGGTGAC p.ValSer54GlyAsp
559838	M5005_RS02895	TOMM family cytolysin streptolysin S, *sagA*	synonymous_variant c.102A>G p.Ser34Ser
592949	M5005_RS03055	ATP-dependent nuclease subunit B, *rexB*	missense_variant c.424A>C p.Lys142Gln
603488	M5005_RS03085	RNA polymerase sigma factor RpoD, *rpoD*	missense_variant c.406G>A p.Ala136Thr
608842	M5005_RS03115	ABC transporter ATP-binding protein	synonymous_variant c.84C>T p.Arg28Arg
645318	M5005_RS03290	carbamoyl phosphate synthase small subunit	missense_variant c.1073C>T p.Thr358Met
687224	M5005_RS03480	phosphomevalonate kinase	missense_variant c.799C>A p.Leu267Ile
717680	M5005_RS03620	DNA topoisomerase IV subunit A, *parC*	synonymous_variant c.2238G>A p.Leu746Leu
768350	M5005_RS03860	phosphoglucosamine mutase, *glmM*	missense_variant c.939C>A p.Asp313Glu
768827	Intergenic		Snp. G>A
779899	M5005_RS09825	nucleoside-diphosphate kinase	missense_variant c.113T>C p.Leu38Pro
782255	Intergenic		Snp. A>G
782340	Intergenic		Del. GAACAA>G
791442	M5005_RS03970	extracellular solute-binding protein	missense_variant c.1034T>A p.Val345Glu
850245	M5005_RS04300	nucleobase:cation symporter-2 family protein	missense_variant c.305A>T p.Lys102Met
882281	M5005_RS04455	methylenetetrahydrofolate--tRNA-(uracil(54)- C(5))-methyltransferase (FADH(2)-oxidizing) TrmFO, *trmFO*	missense_variant c.847G>A p.Ala283Thr
910453	M5005_RS04590	ABC-F family ATP-binding cassette domain-containing protein	stop_gained c.186G>A p.Trp62*
937373	M5005_RS04720	HAMP domain-containing sensor histidine kinase	synonymous_variant c.393A>G p.Gly131Gly
942163	M5005_RS04735	phosphate signaling complex protein PhoU, *phoU*	missense_variant c.11C>T p.Thr4Met
957886	Intergenic		Snp. T>C
959346	Intergenic		Snp. C>T
1003656	M5005_RS05075	minor capsid protein	frameshift_variant c.358dupA p.Ile120fs
1046996	M5005_RS05320	D-alanine--poly(phosphoribitol) ligase subunit DltA, *dltA*	missense_variant c.193A>G p.Asn65Asp
1064592	M5005_RS05405	pseudouridine synthase	synonymous_variant c.705G>A p.Leu235Leu
1121024	M5005_RS05665	hypothetical protein	hypothetical protein

1147752	M5005_RS05790	glucosaminidase domain-containing protein	glucosaminidase domain-containing protein
1191744	M5005_RS06105	deoxyribodipyrimidine photo-lyase	deoxyribodipyrimidine photo-lyase
1212101	M5005_RS06200	translational GTPase TypA, *typA*	translational GTPase TypA
1219790	M5005_RS06240	LacI family DNA-binding transcriptional regulator	LacI family DNA-binding transcriptional regulator
1233554	M5005_RS06310	MmcQ/YjbR family DNA-binding protein	MmcQ/YjbR family DNA-binding protein
1243436	M5005_RS06360	type I-C CRISPR-associated protein Cas8c/Csd1, *cas8c*	frameshift_variant c.1524delC p.Tyr509fs
1244515	M5005_RS06360	type I-C CRISPR-associated protein Cas8c/Csd1, *cas8c*	frameshift_variant c.439_445delGATTCTT p.Asp147fs
1257716	M5005_RS06430	shikimate dehydrogenase	missense_variant c.510G>T p.Lys170Asn
1262625	M5005_RS06440	response regulator transcription factor	missense_variant c.659C>T p.Thr220Ile
1294671	Intergenic		Del. CT>C
1311463	M5005_RS06665	LysR family transcriptional regulator	missense_variant c.376G>A p.Val126Ile
1352751	M5005_RS06850	type 1 glycerol-3-phosphate oxidase, *glpO*	missense_variant c.676G>A p.Ala226Thr
1355235	Intergenic		Snp. C>T
1369715	M5005_RS06925	tagatose-bisphosphate aldolase, *lacD*	missense_variant c.172G>A p.Glu58Lys
1391334	M5005_RS07050	gp58-like family protein	synonymous_variant c.252T>G p.Ala84Ala
1415991	M5005_RS07185	phage/plasmid primase, P4 family	synonymous_variant c.450C>T p.Val150Val
1434386	Intergenic		Ins. A>AT
1458349	M5005_RS07445	histidine phosphatase family protein	missense_variant c.563T>C p.Leu188Pro
1467405	M5005_RS07495	universal stress protein	missense_variant c.86T>C p.Val29Ala
1488191	M5005_RS07570	heme-binding protein Shr, *shr*	synonymous_variant c.486T>C p.Gly162Gly
1547670	Intergenic		Snp. T>C
1568351	M5005_RS07960	DNA-directed RNA polymerase subunit delta, *rpoE*	synonymous_variant c.564A>G p.Glu188Glu
1624722	M5005_RS08250	NADPH-dependent FMN reductase	synonymous_variant c.123T>C p.Asp41Asp
1630160	Intergenic		Del. CT>C
1636851	M5005_RS08295	pullulanase	synonymous_variant c.2943T>C p.Ala981Ala
1681273	M5005_RS08470	complement-mediated lysis inhibitor Sic, *sic*	disruptive_inframe_deletion c.666_674delCTTTGATGG p.Phe223_Gly225del
1732470	M5005_RS08710	urocanate hydratase	synonymous_variant c.21C>T p.Thr7Thr
1751874	M5005_RS08770	alpha,alpha-phosphotrehalase, *treC*	synonymous_variant c.75G>A p.Val25Val
1795728	M5005_RS08990	YhgE/Pip domain-containing protein	missense_variant c.790A>G p.Asn264Asp
1804108	M5005_RS09030	DHH family phosphoesterase	missense_variant c.10T>A p.Phe4Ile
1809755	M5005_RS09055	L-serine ammonia-lyase, iron-sulfur-dependent, subunit alpha, *sdaAA*	synonymous_variant c.330T>C p.Val110Val
1826928	Intergenic		Ins. A> ATTAATCAAAAAATTCATTTGGGTCTTATTT

*Note: Alignments were referenced from NCBI Accession NC_007297.

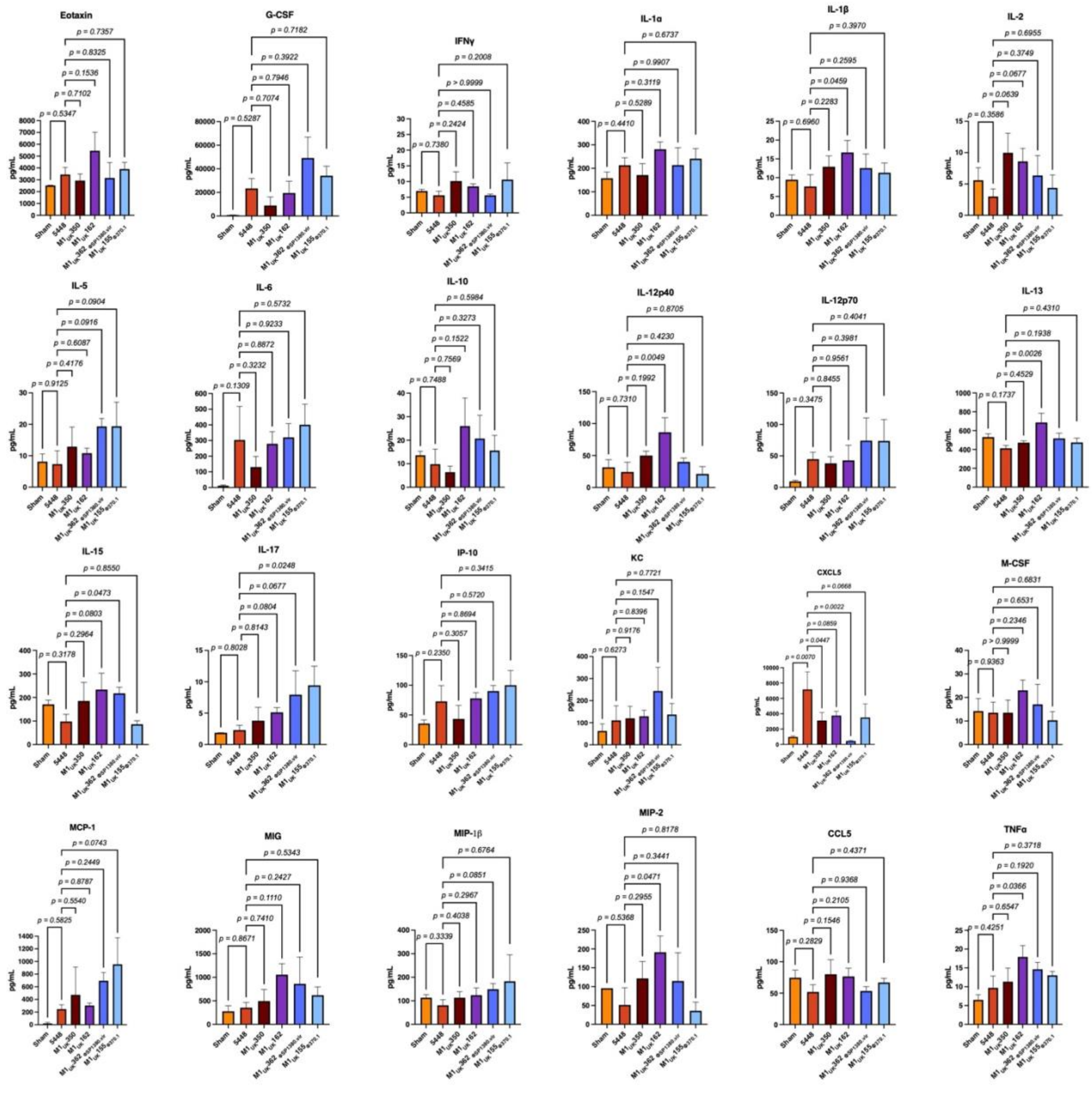

Figure S1. Raw data values of cytokines and chemokines profiles after 48 hours of skin infection with 5448 and $M1_{UK}$ strain. Ordinary one-way ANOVA was performed with n=4 per group and the data is presented as the mean +/- SE. A *p*-value <0.05 was considered statistically different.
